# Left Ventricular Rotational Abnormalities in Treated Hypopituitarism: Insights From the Three-Dimensional Speckle-Tracking Echocardiographic MAGYAR-Path Study

**DOI:** 10.3389/fcvm.2021.703146

**Published:** 2021-10-27

**Authors:** Árpád Kormányos, Nándor Gyenes, Ágnes Horváth, Nóra Ambrus, Csaba Lengyel, Zsuzsanna Valkusz, Attila Nemes

**Affiliations:** Department of Medicine, Medical Faculty, Albert Szent-Györgyi Clinical Center, University of Szeged, Szeged, Hungary

**Keywords:** three dimensional, echocardiography, hypopituitarism, speckle-tracking, left ventricular, rotational mechanics

## Abstract

**Introduction:** Hypopituitarism is a rare, often underdiagnosed, complex hormonal disease caused by the decreased secretion of one or more hormones in the pituitary gland. The present study was designed to assess left ventricular (LV) rotational mechanics in patients with treated hypopituitarism. It was also aimed to find possible differences in these parameters according to the origin of hypopituitarism (congenital vs. acquired).

**Methods:** The present prospective study involved 35 treated patients with hypopituitarism; however, 4 patients had to be excluded due to inferior image quality. The mean age of the remaining 31 cases was 56.3 ± 13.2 years (18 males). The control group consisted of 29 age- and sex-matched healthy volunteers (mean age: 55.3 ± 4.8 years, 14 males). In all cases a complete two-dimensional echocardiography examination was performed followed by three-dimensional speckle-tracking echocardiography.

**Results:** No significant differences could be found in LV volumes between the controls and patients with hypopituitarism and hypopituitary subgroups. LV apical rotation (8.1 ± 5.1° vs. 10.6 ± 3.5°, *p* < 0.05) and LV twist (11.9 ± 5.3° vs. 15.1 ± 3.8°, *p* < 0.05) were impaired in the hypopituitary group with normally directed LV rotational mechanics as compared to the healthy controls. However, 13% of patients showed a near absence of LV twist called LV “rigid body rotation” (LV-RBR). There were no significant differences regarding LV apical and basal rotations and twist between acquired and congenital hypopituitary subgroups.

**Conclusions:** Impaired LV apical rotation and twist could be demonstrated in hypopituitarism regardless of its origin. In the present study with small number of patients with hypopituitarism, LV-RBR was present in 13% of cases.

## Introduction

Hypopituitarism is a rare, often underdiagnosed, complex hormonal disease caused by the reduced secretion of one or more hormones in the pituitary gland. The disease can manifest after birth, called congenital hypopituitarism, or more often, it can be acquired due to vascular, inflammatory, or infectious origin or other causes (1, 2). Due to the disease being hormonal in nature, clinical symptoms may vary heavily based on the type of hormonal deficiency entailing a wide range of possible cardiovascular comorbidities (2, 3). The most frequent causes for vascular mortality in hypopituitarism are stroke, myocardial infarction, and consequent heart failure (2, 4). Early identification of changes in left ventricular (LV) mechanics could help in early identification of patients at risk.

Echocardiography is the main and most affordable tool of the cardiologists to non-invasively assess the heart. Recent advancements in echocardiographic imaging made the three-dimensional (3D) volumetric and strain imaging of the heart possible; out of the novel tools, 3D speckle-tracking echocardiography (STE) is especially useful. 3DSTE provides a possibility for clinicians to assess LV volumes and strains, and for quantification of LV rotational mechanics at the same time using the same digitally acquired 3D datasets (5–9). The present study was designed to assess LV rotational mechanics in patients with treated hypopituitarism. It was also aimed to find possible differences in these parameters according to the origin of hypopituitarism (congenital vs. acquired).

## Patients and Methods

### Patient Population

The present prospective study involved 35 treated patients with hypopituitarism; however, 4 patients had to be excluded due to inferior image quality. The mean age of the remaining 31 cases was 56.3 ± 13.2 years (18 males). Hypopituitary patients were treated and cared for by the Division of Endocrinology and Diabetes Mellitus, Department of Medicine, University of Szeged. The control group consisted of 29 age- and sex-matched healthy volunteers (mean age: 55.3 ± 4.8 years, 14 males). Subjects were considered healthy in the absence of classic risk factors, known and/or treated disease, and drug use without electrocardiographic and echocardiographic abnormalities. The hypopituitary group was further classified into two subgroups based on whether hypopituitarism was congenital or acquired. The hormone levels of all patients were within their sex- and age-specific reference ranges at the time of the echocardiographic examinations. None of the enrolled hypopituitary patients had any previous major cardiovascular event (myocardial infarction or stroke of any type). In all cases, a complete two-dimensional (2D) Doppler echocardiography was performed as per the current clinical standards followed by a complete 3DSTE data acquisition (10). Detailed 3DSTE analysis was completed later offline. The study is a part of the Motion Analysis of the heart and *G*reat vessels b*Y* three-dimensional speckle-t*R*acking echocardiography in *Path*ological cases (MAGYAR-Path) Study, which was organized at our department to assess 3DSTE-derived LV rotational mechanics in different disorders among others. “Magyar” means “Hungarian” in Hungarian language. According to the regulations of the institutional human research committee of the University of Szeged, all patients and volunteers gave informed consent. The present study complied with the ethical guidelines set by the 1975 Declaration of Helsinki and all its updated versions.

### Two-Dimensional Echocardiography

For the 2D Doppler echocardiography, a Toshiba Artida^TM^ system (Toshiba Medical Systems, Tokyo, Japan) was used together with an attached PST-30SBP (1–5 MHz) phased-array transducer. All measurements were based on and strictly followed the current chamber quantification guidelines (10). Valvular regurgitation was assessed on a 1–4 subjective visual scale, while valvular stenosis was excluded with Doppler echocardiography.

### Three-Dimensional Speckle-Tracking Echocardiography

**Figure 1:**
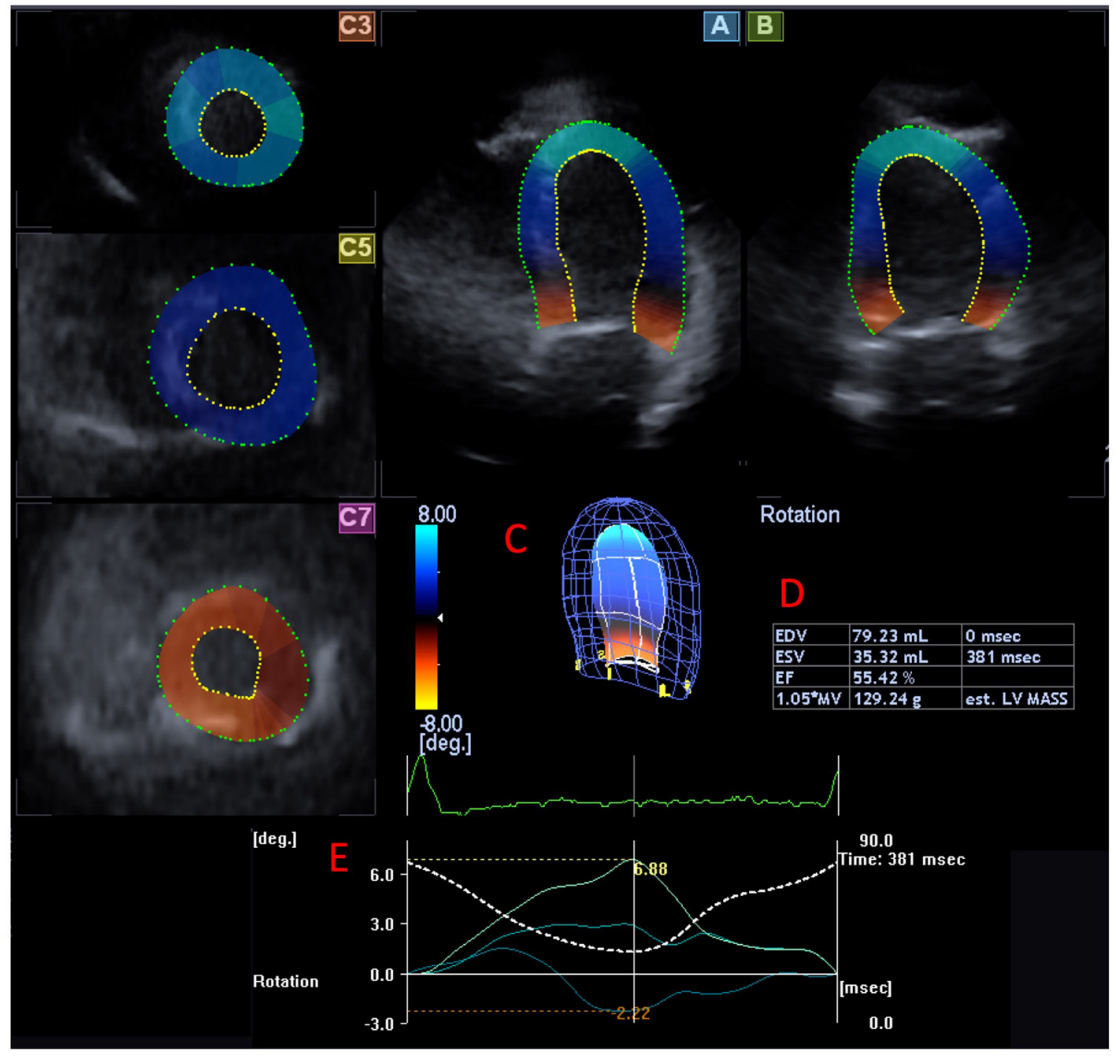
Three-dimensional (3D) speckle-tracking measurement of left ventricular (LV) rotational mechanics from an LV focused view in a patient with hypopituitarism is presented. Apical four-chamber **(A)** and two-chamber views **(B)** and apical (C3), midventricular (C5), and basal (C7) short-axis views are presented extracted from the acquired 3D volumetric dataset are presented. **(C)** Shows the software-generated LV cast, while **(D)** presents LV volumetric parameters. **(E)** Section shows the LV rotation curves and corresponding peak values at the apical, midventricular, and basal LV levels (colored lines). Dashed line represents LV volumetric changes during the cardiac cycle.

3DSTE was performed with the same aforementioned Toshiba Artida^TM^ imaging equipment (Toshiba Medical Systems, Tokyo, Japan) attached to a PST-25SX matrix-array transducer. During image acquisition, a full volume, pyramid-shaped dataset was obtained from the apical window during six constant RR intervals and breath holding (5, 8). Offline image analysis was performed using the vendor-provided 3D Wall Motion Tracking software version 2.7 (Ultra Extend, Toshiba Medical Systems, Tokyo, Japan). During measurements, the operator selects suitable apical four-chamber and two-chamber views and then sets up the cross-sectional planes at LV base, midventricular segments, and lastly at the apex. These steps are followed by semi-automatic endocardial border detection. Based on the input data, the software creates a virtual 3D cast of the LV following a sequential analysis (Figure 1). Based on the virtual 3D cast of the LV, the software is able to calculate regional LV rotational parameters such as clockwise basal and counterclockwise apical LV rotations, LV twist (which is the net difference of LV apical and basal rotations), and time-to-peak LV twist. If basal and apical LV rotations were similarly directed (clockwise or counterclockwise), LV moves like a rigid body without a twisting mechanism, which explains why this sort of movement is called LV “rigid body rotation” (LV-RBR). The net difference of LV basal and apical rotations at peak systole defined by ECG in these cases is called LV apico-basal gradient.

### Statistical Analysis

All statistical data are reported as mean ± standard deviation format for continuous variables; for categorical variables, number and percentage format was used. *p* < 0.05 was considered to be statistically significant. Fischer's exact test was used for all categorical variables. Normal distribution testing was performed using Shapiro-Wilks test; for assessing homogeneity of variance, Levene's test was utilized. Student's two-tailed *t*-test was used in the presence of normal distribution; in case of non-normal distribution, Mann–Whitney–Wilcoxon test was performed. Intraobserver and interobserver variability were assessed by intraclass correlation coefficient (ICC) determination. Statistical analyses were performed with an RStudio (11). For offline data analysis and graph creation, MATLab was used (The MathWorks Inc, Natick, Massachusetts).

## Results

### Demographic Data

All hypopituitary patients had combined hormonal deficiencies: growth hormone (GH) deficiency was present in 29/31 (94%), central hypothyroidism was present in 12/31 (39%), hypogonadotropic hypogonadism was present in 12/31 (39%), central adrenal insufficiency was present in 26/31 (84%), and diabetes insipidus was present in 5/31 (16%) patients.

From standard demographic data, only hypertension proved to be more frequent in the hypopituitary group; all the other risk factors showed no significant differences between the groups (Table 1).

**Table 1:** Demographic and clinical data of patients with hypopituitarism and controls.

	**Controls** **(***n*** = 29)**	**Hypopituitary patients** **(***n*** = 31)**	***P*** **-value**
**Risk factors**			
Age (years)	55.3 ± 4.8	56.3 ± 13.2	0.7
Male sex (%)	14 (48)	18 (58)	0.6
BMI (kg/m^2^)	28.1 ± 4.4	28.8 ± 4.9	0.6
BSA (m^2^)	1.9 ± 0.2	1.7 ± 0.7	0.2
Hypertension (%)	0 (0)	16 (52)	<0.0001
Hypercholesterolemia (%)	0 (0)	4 (13)	0.1
Diabetes mellitus (%)	0 (0)	2 (6)	0.5

*BMI, body mass index; BSA, body surface area*.

### Two-Dimensional Doppler Echocardiography

LV posterior wall was significantly thicker and LV end-systolic diameter and body surface area (BSA)-indexed LV end-systolic diameter were significantly smaller in patients with hypopituitarism compared to controls; all the other routine 2D echocardiographic parameters showed no significant differences (Table 2). None of the healthy subjects or patients showed more than grade 1 valvular regurgitation or had significant stenosis on any valves.

**Table 2:** Two-dimensional echocardiographic data of hypopituitary patients and controls.

	**Controls** **(***n*** = 29)**	**Hypopituitary patients** **(***n*** = 31)**	***P*** **-value**
**Two-dimensional echocardiography**			
LA diameter in PLA (mm)	39.4 ± 3.6	38.8 ± 5.0	0.7
BSA-indexed LA diameter (mm/m^2^)	20.5 ± 2.4	17.2 ± 7.6	0.09
LV end-diastolic diameter (mm)	48.4 ± 2.8	48.8 ± 3.1	0.5
BSA-indexed LV end-diastolic diameter (mm/m^2^)	25.3 ± 2.7	21.6 ± 9.6	0.1
LV end-diastolic volume (ml)	110.5 ± 18.6	114.6 ± 22.8	0.4
BSA-indexed LV end-diastolic volume (ml/m^2^)	59.1 ± 7.6	48.6 ± 25.7	0.1
LV end-systolic diameter (mm)	32.0 ± 2.5	30.1 ± 2.9	0.01
BSA-indexed LV end-systolic diameter (mm/m^2^)	16.7 ± 2.4	13.3 ± 6.0	0.03
LV end-systolic volume (ml)	39.3 ± 7.5	38.4 ± 11.5	0.7
BSA-indexed LV end-systolic volume (ml/m^2^)	20.6 ± 3.4	16.1 ± 9.3	0.06
Interventricular septum (mm)	9.5 ± 1.3	10.0 ± 1.0	0.06
LV posterior wall (mm)	9.5 ± 1.6	10.0 ± 0.9	0.03
E (cm/s)	67.0 ± 17.0	73.9 ± 18.1	0.1
A (cm/s)	71.6 ± 17.9	73.9 ± 16.9	0.6
E/A	0.97 ± 0.25	1.06 ± 0.38	0.6
LV ejection fraction (%)	64.3 ± 3.6	65.9 ± 5.6	0.6

*LA, center atrium; PLA, parasternal long-axis view; BSA, body surface area; LV, center ventricle; E, early transmitral flow velocity; A, late transmitral flow velocity*.

### Three-Dimensional Speckle-Tracking Echocardiography

No significant differences could be found in LV volumes between the controls and patients with hypopituitarism and hypopituitary subgroups. The near absence of LV twist, so-called LV-RBR, could be demonstrated in 4/31 (13%) patients with treated hypopituitarism. Their data were managed separately from the patients with normally directed LV rotational mechanics (*n* = 27). LV-RBR could not be detected in healthy control subjects. In the remaining patients with hypopituitarism, LV apical rotation (8.1 ± 5.1° vs. 10.6 ± 3.5°, *p* = 0.04) and LV twist (11.9 ± 5.3° vs. 15.1 ± 3.8°, *p* = 0.01) were impaired as compared to the healthy controls. There were no significant differences regarding LV apical and basal rotations and twist between acquired and congenital hypopituitary subgroups. The average frame rate of the measurements was 28.1 ± 1.3 vps. 3DSTE-derived LV volumetric, LV rotational, and twist data are presented in Table 3.

**Table 3:** Comparison of three-dimensional speckle-tracking echocardiography-derived left ventricular volumetric and rotational parameters between those of patients with hypopituitarism and controls.

	**Controls** **(***n*** = 29)**	**Hypopituitary** **patients without LV-RBR** **(***n*** = 27)**	**Congenital hypopituitary patients without LV-RBR** **(***n*** = 14)**	**Acquired hypopituitary patients without LV-RBR** **(***n*** = 13)**
**LV volumetric parameters**				
EDV (ml)	82.4 ± 20.5	79.5 ± 29.7	79.1 ± 28.2	80.0 ± 31.9
ESV (ml)	35.6 ± 11.1	34.9 ± 18.1	33.7 ± 15.5	36.0 ± 20.6
EF (%)	57.1 ± 6.8	57.9 ± 8.5	58.3 ± 7.1	57.5 ± 9.8
**LV rotational parameters**				
Basal rotation (degrees)	−4.5 ± 2.5	−3.8 ± 2.0	−4.3 ± 2.1	−3.2 ± 1.8
Apical rotation (degrees)	10.6 ± 3.5	8.1 ± 5.1^*^	7.7 ± 4.6	8.5 ± 4.9
Twist (degrees)	15.1 ± 3.8	11.9 ± 5.3^*^	12.0 ± 5.1	11.8 ± 5.7
Time to peak twist (ms)	323 ± 51	346 ± 114	324 ± 79	369 ± 142

*EDV, end-diastolic volume; ESV, end-systolic volume; EF, ejection fraction; LV-RBR, center ventricular “rigid body rotation*.”

* *p < 0.05 vs. controls*.

To further assess the effects of hypertension on the results, hypopituitary patients were delineated based on the presence of hypertension into two subgroups. LV twist was significantly reduced in hypopituitary patients regardless of the presence or absence of hypertension as compared to controls. Data are presented in Table 4.

**Table 4:** Comparison of three-dimensional speckle-tracking echocardiography-derived left ventricular rotational parameters between those of hypopituitary patients with hypertension or without hypertension and controls.

	**Controls** **(***n*** = 29)**	**Hypopituitary patients without LV-RBR** **without hypertension** **(***n*** = 12)**	**Hypopituitary patients without LV-RBR** **with hypertension** **(***n*** = 15)**
**LV rotational parameters**			
Basal rotation (degrees)	−4.5 ± 2.5	−4.3 ± 2.1	−3.3 ± 1.9
Apical rotation (degrees)	10.6 ± 3.5	7.6 ± 4.3^*^	8.5 ± 5.7
Twist (degrees)	15.1 ± 3.8	11.9 ± 4.8^*^	11.9 ± 5.9^*^
Time-to-peak twist (ms)	323 ± 51	400 ± 120^*^	303 ± 90

*LV-RBR, center ventricular “rigid body rotation*.”

* *p < 0.05 vs. controls*.

### Patients Showing Left Ventricular Rigid Body Rotation

In two cases, counterclockwise LV-RBR could be detected with mean LV basal and apical rotations and LV apico-basal gradient of 4.7 ± 1.1°, 7.3 ± 2.8°, and 2.6 ± 1.6°, respectively [mean age: 60.0 ± 18.4 years, 1/2 (50%) males, 1/2 (50%) acquired hypopituitarism, 1/2 (50%) hypertension, no diabetes mellitus or hyperlipidemia]. In two other cases LV-RBR proved to be clockwise-oriented with mean LV basal and apical rotations and LV apico-basal gradient of −3.2 ± 3.4°, −1.7 ± 0.7°, and 1.4 ± 4.1°, respectively [mean age: 67.5 ± 3.5, 1/2 (50%) males, all acquired hypopituitarism, 1/2 (50%) hypertension, no diabetes mellitus or hyperlipidemia].

### Intraobserver and Interobserver Variability Analysis

Intraobserver ICCs were 0.85, 0.82, and 0.84 for basal and apical LV rotations and LV twist, respectively. Interobserver ICCs proved to be 0.84, 0.80, and 0.81 for the same parameters, respectively.

## Discussion

The main findings of the present study are that LV apical rotation and twist are impaired in patients with hypopituitarism regardless of the disease being acquired or congenital in nature. LV-RBR is also present in the study population (13%). Hypopituitarism is defined as a deficiency of one or more of the hormones secreted by the pituitary gland. The pituitary hormones play a vital role in regulating endocrine function within the body. Hypopituitarism is acquired in most of the cases; however, due to different genetic discrepancies, the disease can manifest in a congenital form as well (1–3). Either congenital or acquired, hypopituitarism is associated with increased morbidity and mortality due to respiratory and cardiovascular diseases; therefore, early diagnosis is important to prevent adverse outcomes. Stroke, myocardial infarction, and consequent heart failure are the most prominent causes for increased cardiovascular mortality in hypopituitarism (2, 4). Early detection of changes in LV volumetric and functional abnormalities could help in selecting patients with increased risk. In recent studies, diagnostic and prognostic impact of LV deformation have been demonstrated in several disorders (12–22). LV has a special 3D movement respecting the cardiac cycle as well due to its special helical 3D structure. LV base and apex rotate in opposite clockwise and counterclockwise directions during the heart cycle resulting in a towel-wringing like motion called LV twist, which is the net difference between rotation of the LV base and apex (9, 23). Its prognostic role is not confirmed but seems to have a role in fine organization of LV function (5, 9, 24). The novel 3DSTE seems to be capable of simultaneously assessing LV volumes, deformation, and rotational mechanics. Factors affecting the endocardium are associated with LV hyperrotation; if the epicardium is affected, LV hyporotation could be detected. Normal reference values of LV rotational parameters are available as well (5, 8, 25–28).

Studies assessing LV deformation and rotational abnormalities are severely lacking in hypopituitarism. Mihaila et al. presented decreased 2DSTE-derived longitudinal (LS) and circumferential strains in the presence of GH deficiency, which was accompanied with impaired LV basal rotation and twist (29). In contrast, increased 3DSTE-derived LS and area strain could be detected in our group of patients with treated hypopituitarism in a recent study, in which most cases had GH deficiency (19). In the present study, reduced LV twist was associated with impaired LV apical rotation and preserved LV basal rotation in patients with normally directed LV rotational mechanics, in which abnormalities were present regardless of the type of hypopituitarism (congenital vs. acquired). To investigate the effect of hypertension on the results, hypopituitary patients were analyzed based on the presence of hypertension. Results also showed reduced LV twist regardless of hypertension. Moreover, 13% of patients showed LV-RBR. The prevalence of LV-RBR have been demonstrated to be high in several disorders like non-compaction cardiomyopathy (50–100%), cardiac amyloidosis (60%), or acromegaly (20%) but were found to be 6% in a normal healthy group of subjects as well (5, 12, 16–18). The other important difference between the study of Mihaila et al. and ours is the method used. According to the guidelines, 2DSTE is not suggested to be used for LV rotational mechanics; 3DSTE seems to be an emerging method due to its nature of seeing and assessing the heart and LV as a 3D organ (5–8).

Although patients were effectively treated with hormone replacement therapy and were in sex- and age-specific reference ranges, their earlier absence or reduced presence could have effects on the presented abnormalities. It can be hypothesized that these changes might partly be the result of GH deficiency as this is directly linked to the amount of cardiac muscle mass (29). Similar abnormalities in acromegaly with higher presence of LV-RBR could be demonstrated in a recent study (16). However, the effects of other hormones could not be excluded either. Moreover, LV hyperfunction of the longitudinal contractility represented by increased LS could be explained by LV rotational abnormalities demonstrated in this study, as a compensatory effect. However, further studies are warranted in this field.

### Limitation Section

The following important limitations have arisen during the assessments:

- Most patients with hypopituitarism had hypertension which could affect the results. However, hypertension is associated with LV hyperrotation (30), which further strengthens our findings.
- Only a limited number of patients with hypopituitarism were assessed. However, hypopituitarism is a rare disease and most patients alive and treated in our tertiary endocrine center have been involved in the present study.
- In the present study, we set out to assess 3DSTE-derived LV apical and basal rotations and twist, although the methodology would enable LV strain measurements as well.
- 3DSTE is known to have lower spatial and temporal resolution compared to 2D echocardiography, which might affect image quality and measurements.
- Due to the validated nature of 3DSTE-derived assessment of LV rotational mechanics, it was not aimed to be performed in this study.

## Conclusions

Impaired LV apical rotation and twist could be demonstrated in hypopituitarism regardless of its origin. In the present study with small number of patients with hypopituitarism LV-RBR was present in 13% of cases.

## Data Availability Statement

The datasets presented in this article are not readily available because restrictions are present due to hungarian regulations. Requests to access the datasets should be directed to nemes@in2nd.szote.u-szeged.hu.

## Ethics Statement

The studies involving human participants were reviewed and approved by Institutional Human Research Committee of University of Szege. The patients/participants provided their written informed consent to participate in this study.

## Author Contributions

ÁK: writing paper and echocardiographic examination. NG: echocardiographic examination. ÁH: patient selection and data collection. NA: data collection. CL: supervision. ZV: supervision and data collection. AN: writing paper and supervision. All authors contributed to the article and approved the submitted version.

## Conflict of Interest

The authors declare that the research was conducted in the absence of any commercial or financial relationships that could be construed as a potential conflict of interest.

## Publisher's Note

All claims expressed in this article are solely those of the authors and do not necessarily represent those of their affiliated organizations, or those of the publisher, the editors and the reviewers. Any product that may be evaluated in this article, or claim that may be made by its manufacturer, is not guaranteed or endorsed by the publisher.
